# Apnea testing for brain death confirmation in VV-ECMO patients with very low sweep flow: a case reports and practical physiological insights

**DOI:** 10.62675/2965-2774.20250373

**Published:** 2025-06-30

**Authors:** Carine Carrijo de Faria, Pedro Vitale Mendes, Luis Carlos Cardoso Maia, Gabriel Afonso Dutra Kreling, Marcelo Park

**Affiliations:** 1 Universidade de São Paulo Faculdade de Medicina Hospital das Clínicas São Paulo SP Brazil Intensive Care Unit, Hospital das Clínicas, Faculdade de Medicina, Universidade de São Paulo - São Paulo (SP), Brazil.

**Keywords:** Apnea, Brain death, Extracorporeal membrane oxygenation, Respiratory insufficiency, Respiration disorders

## Abstract

In recent years, venovenous extracorporeal membrane oxygenation has become a critical therapeutic tool for patients with severe respiratory failure. Neurological complications, including brain death, are common in this population, and confirming brain death in venovenous extracorporeal membrane oxygenation-supported patients presents unique challenges. In Brazil, an apnea test is mandatory for confirming brain death. However, its application in patients on venovenous extracorporeal membrane oxygenation, which predominantly addresses venoarterial extracorporeal membrane oxygenation cases, is not well defined in the literature. This report outlines our standardized approach for conducting apnea tests in three patients with suspected brain death during ongoing venovenous extracorporeal membrane oxygenation support. We describe three cases from a cohort of 93 extracorporeal membrane oxygenation patients treated for severe respiratory failure. The apnea test was conducted after 24 hours of observation without sedation. Given the physiological nuances of extracorporeal membrane oxygenation, where carbon dioxide clearance is primarily influenced by sweep flow, we adopted a low-sweep-flow protocol (200mL/minute) to achieve a partial pressure of carbon dioxide greater than 55mmHg, consistent with brain death criteria. In cases of severe hypoxemia during the test, extracorporeal membrane oxygenation blood flow can be temporarily increased to maintain oxygenation. All patients received concurrent renal support, which also facilitated carbon dioxide clearance. Our findings suggest that the apnea test with very low sweep flow is a safe and feasible method for diagnosing brain death in venovenous extracorporeal membrane oxygenation-supported patients. This physiologically grounded approach provides a clinically viable strategy for managing the complex interplay between gas exchange, oxygenation, and carbon dioxide clearance during the apnea test.

## INTRODUCTION

Venovenous extracorporeal membrane oxygenation (VV-ECMO) support for patients with severe respiratory failure has increased in recent decades.^(1)^ Neurological complications are common in this population,^(2)^ and brain death is occasionally observed.^(2,3)^ In Brazil, the apnea test is mandatory for confirming brain death,^(4)^ but performing it in patients supported by VV-ECMO is not straightforward. The interplay among disease severity, organ support, and VV-ECMO gas exchange makes carbon dioxide (CO_2_) kinetics particularly complex. Furthermore, most of the current apnea testing literature focuses on venoarterial extracorporeal membrane oxygenation (VA-ECMO).^(5)^ Therefore, we designed a physiologically grounded apnea test for three VV-ECMO-supported patients. Here, we report and discuss our standardized experience.^(3)^

### Case description

Informed consent was waived by the Research Ethics Committee of the *Hospital das Clinics* of the *Universidade de São Paulo*, due to the observational nature of this study (registration 107.443).

Since 2011, 93 patients have received ECMO at our institution. Of these, 43/93 (46%) died during hospitalization. Among these, 7/43 (16%) were diagnosed with brain death (four after decannulation: 2-VV-ECMO, 1-VA-ECMO, and 1 following venoarterial-venous [VAV]-ECMO). Three patients who progressed to brain death while receiving VV-ECMO support were included in this study. All were referred to our hospital and managed with VV-ECMO support. Suspicion of brain death arose during routine hourly patient evaluation by the multiprofessional team. Clinical signs included fixed, dilated, and nonreactive pupils; absence of motor responses despite low or no sedation at the time of respiratory support; and the absence of respiratory movements.

**Figure 1:**
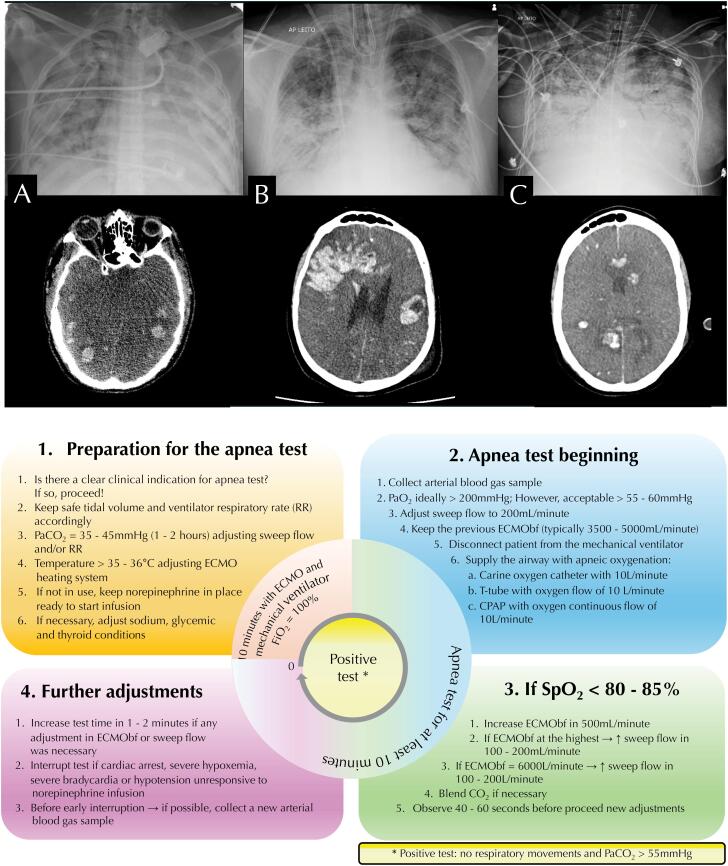
Thoracic and cerebral images of the patients (Panels A, B, and C) and the structure of our standardized apnea test during ongoing venovenous extracorporeal membrane oxygenation support (Panel D).

**Patient 1 -** Admitted with a diagnosis of severe community-acquired pneumonia without any comorbidity and developed severe hypoxemic respiratory failure. Prone positioning and alveolar recruitment were unsuccessful. Bronchopleural fistula and multiple organ failure (MOF) complicated his clinical status. Brain death was suspected two days after VV-ECMO initiation. Additional clinical details are provided in table 1 and figure 1A show further patients’ characteristics. This case was previously reported, at which time two apnea tests were required in Brazil.^(3)^ During the first test, oxygen saturation of 77% was reached in the final arterial blood gas analysis; however, the peripheral oxygen saturation (SpO_2_) remained above 80%.

ECMO - extracorporeal membrane oxygenation; PaO_2_ - partial pressure of oxygen; ECMObf - extracorporeal membrane oxygenation blood flow; CPAP - continuous positive airway pressure; FiO2 - fraction of inspired oxygen; CO_2_ - carbon dioxide; PaCO_2_ - partial pressure of carbon dioxide.

**Table 1:** Clinical and laboratorial characteristics of the three patients prior to initiation of venovenous extracorporeal membrane oxygenation support and during the apnea test

		Patient 1				Patient 2		Patient 3	
Age (years)		32				34		31	
Sex		Male				Male		Female	
SAPS 3		74				81		93	
Weight (kg)		84				85		60	
Height (cm)		191				180		162	
Admission SOFA		8				16		16	
Before ECMO initiation									
	Heart rate (BPM)	135				119		127	
	Mean ABP (mmHg)	80				78		66	
	Norepinephrine (mcg/kg/minute	-----				0.6		1	
	Vasopressin (IU/minute)	-----				0.04		0.04	
	Temperature (°C)	38.0				37.8		36.0	
	BUN/creatinine (mg/Dl)	77/1.76				105/9.17		42/4.59	
	ALT/AST (IU/mL)	159/481				107/197		5,941/2,592	
	Hemoglobin (g/dL)	7.9				10		6.4	
	Leukocytes (cels/mm^3^)	12,770				14,450		19,360	
	Platelets (units/mm^3^)	173,000				209,000		50,000	
	INR	1.89				2.02		1.44	
	RASS	- 5				- 5		- 5	
	Midazolam (mg/kg/hour)	0.25				0.2		0.3	
	Propofol (mg/kg/hour)	-----				0.3		0.2	
	Fentanyl (mcg/hour)	50				40		20	
	Cisatracurium (mg/kg/hour)	0.1				0.2		-----	
	Rocuronium (mg/kg/hour)	-----				-----		0.2	
	Ventilatory mode	PCV				PCV		VCV	
	Respiratory rate (IPM)	38				40		35	
	Tidal volume (mL)	> 300				400		380	
	Plateau pressure (cmH_2_O)	> 32				34		40	
	PEEP (cmH_2_O)	17				14		3	
	FiO_2_ (%)	100				100		100	
	pH	7.06				7.01		7.10	
	PaO_2_ (mmHg)	43				47		35	
	PaCO_2_ (mmHg)	142				87.7		50	
	SBE (mEq/L)	2				-2		-16	
	SatO_2_ (%)	52				76		68	
	P/F ratio (mmHg)	43				47		35	
	Rescue maneuvers	TGI/Prone/Recruitment				Prone		Prone/Recruitment	
	Renal support	CVVH				CVVHDF		CVVHDF	
Apnea test		Before	After	Before	After	Before	After	Before	After
	Airway support	T-tube with oxygen 10L/minute		T-tube with oxygen 10L/minute		T-tube with oxygen 10L/minute		CPAP with oxygen 10L/minute*	
	Heart rate (BPM)	94	95	87	90	115	133	120	124
	Mean ABP (mmHg)	84	85	80	84	107	105	93	113
	Norepinephrine (mcg/kg/minute)	0.15	0.15	-----	-----	0.12	0.12	0.02	0.02
	Vasopressin (IU/minute)	0.03	0.03	0.03	0.03	-----	-----	0.04	0.04
	Temperature (°C)	36.5	36.5	36.5	36.5	36.5	36.4	36.4	36.4
	pH	7.30	7.07	7.32	7.14	7.45	7.12	7.45	7,21
	PaCO_2_ (mmHg)	50.3	87.3	44.4	73.0	35.4	99.6	45	99
	PaO_2_ (mmHg)	67	61.2	100.3	66.3	91.9	58.9	74	70
	SBE (mEq/L)	-2.6	-7.5	-3.9	-6.3	4.2	0.2	8.2	10.1
	SatO_2_ (%)	90.5	77.1	97.0	84.2	95.2	86	97.4	89.1
	ECMO blood flow (mL/minute)	4,500	4,500	4,500	4,500	4,740	4,740	4,310	4,310
	Sweep gas flow (mL/minute)	8,000	200	8,000	200	5,000	200	4,000	200
	ECMO FiO_2_ (%)	100	100	100	100	100	100	100	100
	ECMO temperature (°C)	36.5	36.5	36.5	36.5	36.5	36.5	36.5	36.5
	Time spent on apnea test (minutes)	10		10		14		16	
	Hemoglobin (g/dL)	7.3		7.3		8.6		7.0	
	Leukocytes (cels/mm^3^)	25,810		25,810		27,160		12,810	
	Platelets (units/mm^3^)	87,000		87,000		138,000		49,000	
	INR	3.01		3.01		2.10		1.33	

SAPS 3 - Simplified Acute Physiology Score 3; SOFA - Sequential Organ Failure Assessment; ECMO - extracorporeal membrane oxygenation; ABP - arterial blood pressure; BUN - blood urea nitrogen; ALT - alanine transaminase; AST - aspartate transaminase; INR - international normalized ratio; RASS - Richmond Agitation-Sedation Scale; PCV - pressure-controlled ventilation; VCV - volume-controlled ventilation; PEEP - positive end-expiratory pressure; PaCO_2_ - partial pressure of carbon dioxide; SBE - standard base excess; SatO_2_ - hemoglobin oxygen saturation; P/F - ratio of the partial pressure of oxygen to the fraction of inspired oxygen; TGI - tracheal gas insufflation; CVVH - continuous venous-venous hemofiltration; CVVHDF - continuous venous-venous hemodiafiltration; CPAP - continuous positive airway pressure; FiO2 - fraction of inspired oxygen.

* CPAP = 10cmH_2_O (using a concentric coil valve).

**Patient 2 -** A patient without comorbidities, admitted with COVID-19 and severe respiratory failure, progressing to MOF. Two days after VV-ECMO initiation, brain death was suspected. Clinical data are shown in table 1 and figure 1B.

**Patient 3 -** Systemic erythematous lupus patient, with musculoskeletal, cutaneous, and hematologic involvement, treated with prednisone 40mg. She was admitted with severe alveolar hemorrhage and developed MOF with severe acute liver failure, requiring plasmapheresis, VV-ECMO, and renal support. Brain death was suspected four days after referral. Additional details are shown in table 1 and figure 1C.

All patients were observed for 24 hours without any sedative administration prior to initiating the brain death protocol. The apnea test was standardized as described in figure 1D. Transcranial Doppler confirmed circulatory collapse in all patients. Due to MOF, no organs were procured, and life support was subsequently withdrawn.

## DISCUSSION

In Brazil, an increase in partial pressure of carbon dioxide (PaCO_2_) from 35 - 45 mmHg to > 55mmHg without spontaneous respiration is currently considered compatible with brain death.^(4)^ During ECMO support, CO_2_ clearance depends primarily on sweep flow and, to a lesser extent, on ECMO blood flow.^(6)^ However, reduced sweep flow to facilitate CO_2_ elevation during the apnea test results in prohibitive hypoxemia.^(6)^ Although oxygenation depends on blood flow, minimal fresh gas from sweep flow is still necessary to supply oxygen.^(6)^ Therefore, current guidelines recommend decreasing sweep flow to 0.5 - 1L/minute.^(5)^

This low sweep flow (0.5 - 1L/minute) still allows a CO_2_-transfer as high as 75mL/minute on an ECMO blood flow of 3,500mL/minute^(6)^ and 45mL/minute on a blood flow of 200 - 400mL/minute.^(7)^ Moreover, all patients received continuous renal support during the apnea test. Hypercapnia increases bicarbonate levels, facilitating both bicarbonate and CO_2_ removal through renal support.^(8)^ This makes it more challenging to achieve a PaCO_2_ > 55mmHg. Therefore, we selected a sweep flow of 200mL/minute.^(3)^

The limited oxygen delivery with this very low sweep flow can result in immediate hypoxemia and an ECMO-blood flow roof effect on oxygen transfer in the oxygenator.^(9)^ However, as a rescue maneuver for severe hypoxemia (SpO_2_ < 80 - 85%) during the apnea test, increasing ECMO-blood flow will increase the oxygen delivery, providing a brief window to finish the test safely. If severe hypoxemia persists, a higher sweep flow will be necessary; however, to reach the targeted CO_2_, a CO_2_ blend with the sweep gas may be required.^(10)^

## CONCLUSION

The apnea test using very low sweep flow in patients receiving ongoing venovenous extracorporeal membrane oxygenation support is physiologically grounded, clinically plausible, and safe. Achieving the carbon dioxide levels recommended by Brazilian guidelines at the end of the apnea test is possible. Increasing extracorporeal membrane oxygenation blood flow can be a temporary rescue strategy in cases of significant hypoxemia (oxygen saturation <80 - 85%).
